# IP-10 for the Diagnosis and Treatment Monitoring of Tuberculosis in Children

**DOI:** 10.3390/diagnostics14020177

**Published:** 2024-01-13

**Authors:** Agnieszka Strzelak, Anna Komorowska-Piotrowska, Agnieszka Borowa, Maria Krasińska, Wojciech Feleszko, Marek Kulus

**Affiliations:** 1 Department of Pediatric Pulmonology and Allergy, Medical University of Warsaw, 63A Zwirki i Wigury Street, 02-091 Warsaw, Poland; 2 Department of Lung Diseases and Tuberculosis for Children and Adolescents, Mazovian Center for Treatment of Lung Diseases and Tuberculosis, Reymonta 83/91 Street, 05-400 Otwock, Poland

**Keywords:** tuberculosis, chemokine CXCL10, *Mycobacterium tuberculosis*, LTBI, TB contact

## Abstract

Purpose: To determine the utility of interferon-gamma-inducible protein 10 (IP-10) for identifying active tuberculosis (TB) and TB infection (TBI) in children in BCG-vaccinated populations, establish its diagnostic performance characteristics, and evaluate changes in IP-10 level during anti-TB chemotherapy. Methods: Concentrations of IP-10 and IFN-γ were measured in QuantiFERON-TB Gold (QFT) supernatants in children with suspected TB or due to recent TB contact. A total of 225 children were investigated: 33 with active TB, 48 with TBI, 83 TB contacts, 20 with suspected TB but other final diagnoses, and 41 controls. In 60 children, cytokine responses were evaluated at a follow-up visit after 2 months of anti-TB treatment. Results: IP-10 expression was significantly higher in infected children (active TB and TBI cases) than in uninfected individuals. IP-10 proved effective in identifying TB infection at its optimal cut-off (>1084.5 pg/mL) but was incapable of differentiating between children with active TB and TBI. Combining IP-10 and IFN-γ increased the QFT sensitivity. IP-10 but not IFN-γ decreased significantly during anti-TB treatment in children with active TB (*p* = 0.003). Conclusion: IP-10 identifies TB infection and declines during anti-TB chemotherapy in children. Incorporating IP-10 into new immunodiagnostic assays could improve TB diagnosis and allow for treatment monitoring.

## 1. Introduction

Childhood tuberculosis (TB), caused by *Mycobacterium tuberculosis* (*Mtb*.), is still an urgent global health issue. In the COVID-19 aftermath, this is even more so than before the pandemic. Due to the COVID-19-related disruptions to TB detection and treatment, an increase in TB incidence and mortality has been reported globally [1]. However, epidemiological estimates of childhood TB burden remain underestimated. This is so for several reasons. Firstly, childhood active TB (ATB) is very difficult to diagnose due to diverse clinical presentations, difficulties in obtaining specimens for microbiological evaluation, and paucibacillary disease [2,3]. Secondly, the diagnostic work-up for tuberculous infection (TBI) lacks the gold standard and routinely used immunological tests, namely, the tuberculin skin test (TST) and interferon gamma (IFN-γ) release assays (IGRAs) have considerable limitations in the pediatric population.

The TST shows cross-reactivity with bacillus Calmette–Guérin (BCG) and the majority of non-tuberculous mycobacteria (NTMs) and has therefore low specificity in BCG-vaccinated and NTM-infected children [4,5]. Commercially available IGRAs, ELISA-based QuantiFERON-TB Gold In-Tube (QFT) and ELISPOT-based T-SPOT.TB, have comparable sensitivity and higher specificity than the TST in identifying *Mtb.* infection. However, they perform less reliably in human immune virus (HIV)-infected individuals and young children, albeit with inconsistent results [6,7,8,9]. Neither test discriminates ATB from TBI, nor are they licensed for ATB diagnosis. Yet, they are widely used to facilitate the diagnosis of ATB, with sensitivity reaching 77–85% [10]. The new version of the QFT test, QuantiFERON-TB Gold Plus, has so far exhibited similar properties to previous-generation IGRAs in children in both ATB and TBI [10].

It is plausible that additional cytokine biomarkers may improve IGRAs’ performance [11,12]. One of the most promising surrogate biomarkers is the interferon-gamma-inducible protein 10 (IP-10), a chemokine expressed primarily by antigen-presenting cells upon stimulation by IFN-γ [13]. In the TB field, IP-10-based assays have emerged as alternatives to IGRAs, with IP-10 being released in 100-fold higher concentrations than IFN-γ. IP-10 has comparable accuracy to IGRAs in identifying *Mtb.* infection in adults and children and may even perform better in HIV-infected individuals and the young [11,13,14,15,16,17,18,19,20]. Additionally, IP-10 has been suggested as a potential biomarker for treatment monitoring, but studies in pediatric populations are scarce [21,22].

The aim of the present study was to assess IP-10 responses in children with symptoms of ATB and in TB contact referrals in a low TB incidence country where BCG vaccination is routinely administered at birth. We intended to examine the potential of IP-10 to differentiate between different stages of *Mtb*. infection, to compare IP-10 with QFT and TST results, and to explore the influence of age and anti-tuberculosis treatment on IP-10 levels.

## 2. Methods

### 2.1. Study Population

For this prospective study, we recruited children aged < 18 years admitted to the Children’s Hospital, Medical University of Warsaw, Poland, and the Mazovian Centre for Treatment of Lung Diseases and Tuberculosis, Otwock, Warsaw Agglomeration, Poland between May 2012 and September 2015. Eligible for participation were (1) children with clinical suspicion of TB and (2) children in recent contact with pulmonary TB (PTB). The exclusion criteria included immunosuppression, previous anti-tuberculosis treatment, and a TST performed within the last 18 months to minimize the risk of boosting. Ethics approval for this study was provided by the Bioethical Committee of the Medical University of Warsaw and written informed consent was obtained from the legal guardian of each participant before enrollment.

### 2.2. Clinical and Laboratory Procedures

Demographics, history, and clinical findings were recorded systematically using a case report form. All participants underwent peripheral blood testing including QFT. Children suspected of ATB and contact tracing referrals underwent standard diagnostic procedures encompassing the TST, QFT, chest X-ray, and, if required, histological and bacteriological evaluation (microscopy, PCR, and culture). Not all participants received TST due to recurrent shortages of purified protein derivatives (PPD). For ethical reasons, we did not perform TST, chest X-ray, or microbiological studies in the control group. All children with ATB received standard combined anti-tuberculosis treatment, and children with TBI and TB contacts aged < 5 years were offered preventive chemotherapy. A subgroup of patients underwent follow-up examination two months after treatment onset.

#### 2.2.1. TST

Following an intradermal injection of 2 tuberculin units (0.1 mL) of RT 23 PPD (Statens Serum Institute, Copenhagen, Denmark) into the left lower arm, the transverse induration was recorded 48–72 h later. The TST was defined as positive at ≥10 mm induration.

#### 2.2.2. QuantiFERON-TB GOLD In-Tube

QuantiFERON-TB GOLD in-tube assay was performed according to the manufacturer’s instructions, the concentration of IFN-γ was determined by using the QFT ELISA, and the results were defined using the manufacturer’s software Cellestis Version 2.50.1 (Cellestis/Qiagen, Carnegie, Australia) [23].

#### 2.2.3. IP-10 Determination

IP-10 concentration was measured in the remaining supernatants from QFT tubes using the Quantikine ELISA Human IP-10 Immunoassay (R&D Systems Europe, Ltd., Abingdon, UK) according to the manufacturer’s instructions. All samples were measured in duplicate. The plasma was diluted 1:10 and samples with values outside the upper limit of the standard curve were diluted and measured again. The lower limit of detection (sensitivity) was calculated to be 2.1 pg/mL. IP-10 concentrations are expressed in pg/mL.

### 2.3. Classification of Participants

Following clinical and diagnostic workup, the patients were assigned to one of the following five categories:

1. ATB diagnosed in symptomatic patients with microbiologically confirmed TB (mATB) (positive microscopy, PCR, or culture result) or in patients fulfilling ≥ two of the following three criteria: (1) clinical symptoms suggestive of ATB, (2) radiological findings consistent with ATB, and (3) a history of contact with PTB, all in conjunction with a positive clinical response to anti-tuberculosis treatment.
2. TBI is defined as a positive TST and/or IGRA result in asymptomatic children with a history of contact with PTB and a normal chest X-ray.
3. TB contacts—asymptomatic children with a history of contact with PTB, not fulfilling the criteria for ATB or TBI.
4. Non-TB—children with clinical suspicion of TB, but with other final diagnoses and a positive response to specific treatment.
5. Controls—asymptomatic children after careful exclusion of TB contact and inflammatory comorbidities and with a negative IGRA result.

For simplicity reasons, the term “*Mtb.-*infected” refers to children with TBI and ATB combined together and “*Mtb.-*uninfected” refers to the TB contacts, non-TB group, and controls combined together.

### 2.4. Statistical Analysis

Data were analyzed using STATISTICA 13.1 (SatSoft, Kraków, Poland) and Medcalc 19.1 (MedCalc Software, Ostend, Belgium). Figures were prepared in GraphPad Prism version 8 (GraphPad Software, San Diego, CA, USA). Data are presented as the medians and interquartile ranges (IQRs). We performed the chi-squared test or Fisher’s exact test for qualitative variables, and the Kruskall–Wallis test or the Mann–Whitney test for quantitative variables. Changes in IFN-γ and IP-10 concentrations during treatment were assessed using the Wilcoxon signed-rank test. The Spearman’s rank test was applied to assess biomarker correlation. The diagnostic performance of IP-10 was assessed using the receiver operating characteristic curve (ROC) analysis and the area under the curve (AUC). The highest values of Youden’s index were used for selecting the cut-off values. The TB antigen and mitogen control concentrations of IP-10 were background-corrected by subtracting the concentration in the negative control tube. All tests were two-sided, and a *p*-value of <0.05 was considered significant.

## 3. Results

### 3.1. Study Participant Characteristics

In total, 263 children were eligible for the study; 19 were excluded due to insufficient blood samples, 2 were excluded due to improper sample handling, 2 were excluded due to insufficient data, 10 were excluded due to technical reasons, and 5 children were excluded because the index case was diagnosed with NTMs and not *Mtb.* infection. A total of 225 children were included in the final analysis: 33 with ATB, 48 with TBI, 83 TB contacts, 20 with a non-TB disease, and 41 controls. The patients’ characteristics are shown in Table 1. The ATB patients were older than the other children (*p* < 0.05), and the TB contacts were younger than the *Mtb.-*infected children and the non-TB group (*p* < 0.01). No other significant demographic differences were noted across the TB classification groups. More than 96% of participants were BCG-vaccinated. Of the 33 ATB patients, 9 (27.3%) had a positive culture result, 13 (39.4%) were admitted with a clinical suspicion of TB, and 20 (60.6%) were admitted due to recent TB contact. All ATB patients achieved the resolution of symptoms on anti-tuberculosis treatment. The non-TB children were diagnosed with pneumonia (n5), respiratory tract infections (n6), other respiratory diseases (n4), and “miscellanea” (n5).

### 3.2. IP-10 Responses in Mtb.-Infected and Uninfected Children

The IP-10 responses in the *Mtb*. antigen-stimulated, unstimulated, and mitogen-stimulated supernatants are presented in Table 2. Upon *Mtb.* antigen stimulation, the expression of IP-10 was augmented significantly in *Mtb.-*infected children only (*p* < 0.0001).

**Figure 1:**
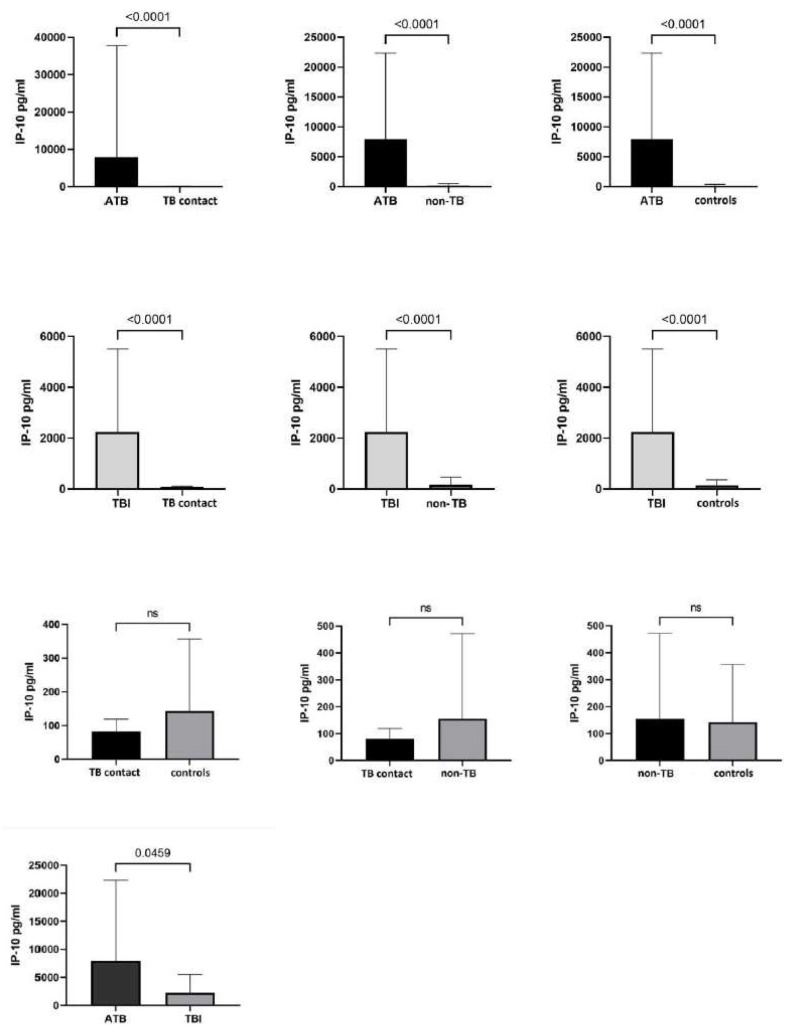
Comparison of Mtb. antigen-stimulated IP-10 responses among the study participants. ATB—active tuberculosis; TBI—tuberculous infection; TB—tuberculosis; ns—non-significant (Mann-Whitney test).

The *Mtb.* antigen-stimulated IP-10 responses were much higher in *Mtb.-*infected children than in *Mtb.*-uninfected children (*p* < 0.0001). We then compared the IP-10 levels between the subgroups of infected and uninfected children (ATB and TBI vs. TB contacts, non-TB, and controls separately), and the differences in the IP-10 levels were still significant (*p* < 0.0001 for all pairwise comparisons) (Figure 1). While the IP-10 level was higher in the ATB group than in the TBI group (*p* = 0.047), no differences were demonstrated among the *Mtb.*-uninfected groups (*p* > 0.05).

### 3.3. IP-10 Identifies Mtb. Infection Regardless of the Reason for Admission

**Figure 2:**
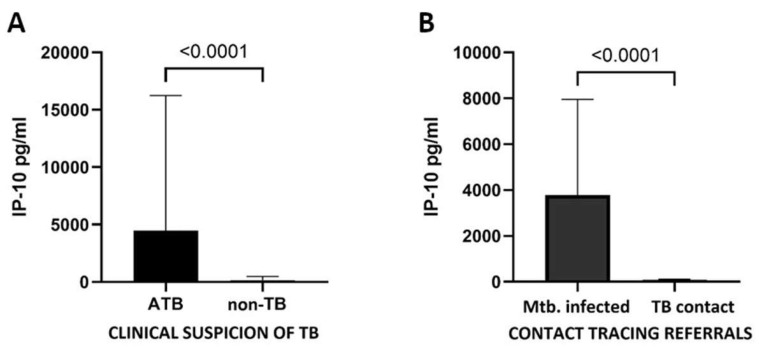
Mtb. antigen-stimulated IP-10 responses in children with clinical suspicion of TB (**A**) and contact tracing referrals (**B**) (Mann–Whitney test).

To reflect daily pediatric clinical practice, we performed a subgroup analysis regarding the reason for hospital admission (Figure 2). In children with clinical suspicion of TB, the IP-10 concentration was significantly higher in the ATB group than in the non-TB group (*p* < 0.0001). Similarly, in contact tracing referrals, *Mtb.-*infected children expressed higher levels of IP-10 than TB contacts (*p* < 0.0001), also when the ATB and TBI groups were compared separately (*p* < 0.0001 for both comparisons).

### 3.4. IP-10 Responses According to the TST, QFT, and Microbiological Test Results

Assuming that Mtb.-infected children constitute a heterogeneous group of patients in terms of the TST, QFT, and microbiological confirmation, we compared children with different test result configurations for IP-10 expression. We found significant differences within the TBI group with higher IP-10 levels in children with TST^+^QFT^+^ TBI than in those with discordant TST and QFT results (TST^−^QFT^+^ and TST^+^QFT^−^; *p* = 0.009 and *p* < 0.0001, respectively). No differences were observed between children with discordance (*p* > 0.05). In the ATB group, there were no differences in IP-10 levels between children with and without microbiological confirmation or with and without positive culture results (*p* = 0.79 and *p* = 0.53, respectively).

### 3.5. IP-10 and IFN-γ Level Are Not Correlated with Age

The *Mtb.* antigen-stimulated concentrations of IP-10 and IFN-γ differed significantly among the four different age groups (0–2 y; 2–5 y; 5–10 y; and >10 y) (*p* < 0.001 and *p* = 0.04, respectively) and between children aged < 5 years and older (*p* < 0.001 and *p* = 0.005, respectively). However, the correlation between biomarker level upon *Mtb.* antigen stimulation and age was weak for IP-10 and negligible for IFN-γ (r_s_ = 0.32; *p* = 0.000001 and r_s_ = 0.16; *p* = 0.014, respectively).

### 3.6. IP-10 Level Decreases during Anti-tuberculosis Treatment

**Figure 3:**
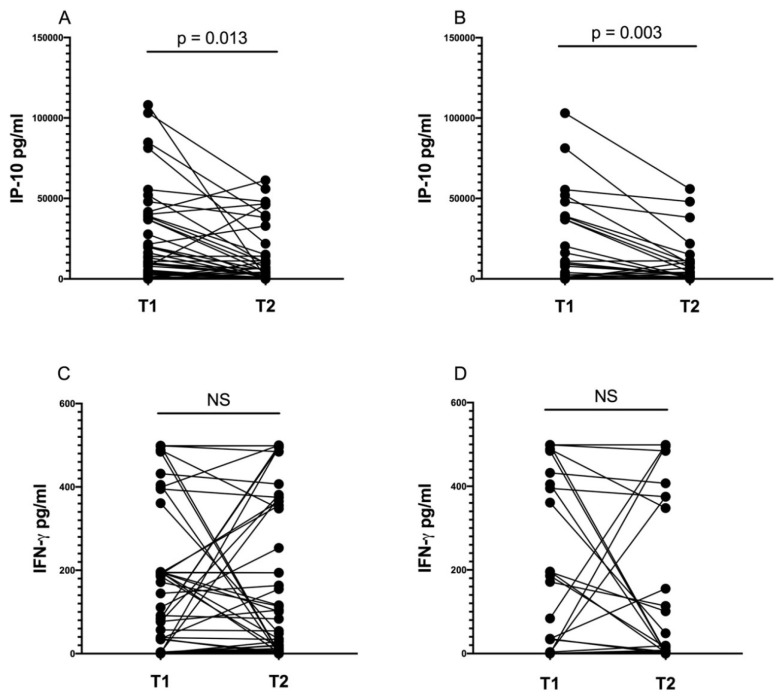
Antigen-stimulated IP-10 (**A**,**B**) and IFN-γ (**C**,**D**) levels before (T1) and after 2 months (T2) of anti-tuberculosis treatment in all controlled children (ATB, TBI, and TB contacts) (**A**,**C**) and only in children with ATB (**B**,**D**) (Wilcoxon signed-rank test).

After two months of anti-tuberculosis therapy (median 69; IQR:64–78.5 days from treatment onset), the IP-10 and IFN-γ levels in the QFT tubes were measured in 25 children with ATB, 29 with TBI, and 6 TB contacts. We noticed a significant decrease in the IP-10 but not IFN-γ level in the whole group after two months of treatment (*p* = 0.0013 and *p* = 0.6, respectively) (Figure 3A,C). This decrease resulted from a significant decline in the IP-10 concentration in the ATB group (Figure 3B,D). No significant decline in IP-10 level was observed in the TBI group (*p* = 0.09).

After the initial phase of chemotherapy, the IP-10 responses still differed significantly between the *Mtb.-*infected and uninfected children (median 1324.4 pg/mL; IQR: 210.9–10,134.9 pg/mL vs. median 111.5 pg/mL; IQR: 0–164.6 pg/mL, *p* = 0.008). IP-10 was significantly higher in children with ATB (median 4139.7 pg/mL; IQR: 1090–10,464.6 pg/mL) than in TB contacts (median 111.5 pg/mL; IQR: 0–164.6 pg/mL) (*p* = 0.0007). However, no significant differences were found between TB contacts and children with TBI (median 658.2 pg/mL; IQR: 36.8–5295.4 pg/mL) (*p* > 0.05). At the follow-up visit, we still observed a significant difference in IP-10 levels between the ATB and TBI groups (*p* = 0.015). Of note, no discrepancies in IFN-γ concentrations were found between children with ATB, TBI, and TB contacts (*p* > 0.05).

### 3.7. Diagnostic Performance of IP-10

Only data from children with microbiologically confirmed ATB, TST^+^QFT^+^ TBI, TST^-^QFT^-^ TB contact, TST^-^QFT^-^ non-TB, and QFT^-^ controls were included in the ROC analysis. For the assessment of IP-10 accuracy in identifying ATB and TBI, data from children with ATB and TBI were compared with the control group. IP-10 showed very good properties for discriminating *Mtb.*-infected children, children with ATB, and children with TBI from the controls. However, IP-10 could not discriminate between the ATB and TBI groups. Additionally, the ROC analysis was performed separately for children admitted due to clinical suspicion of TB and for contact tracing referrals. In both settings, we observed good performance of IP-10 in identifying *Mtb.*-infected children. At its optimal cut-off values, IP-10 showed very good performance in each setting (AUC > 0.9), except for ATB vs. TBI (Table 3).

We then evaluated the properties of the optimal cut-off point of 1084.5 pg/mL for identifying *Mtb.* infection (ATB and TBI combined vs. the control group). At this cut-off, IP-10 correctly classified 84.9% of the participants, 93.3% among the microbiologically confirmed ATB group, and showed 88.5% positive predictive value and 83.5% negative predictive value. IP-10 showed good agreement with both the TST and QFT (85.4% and 87.7% respectively; k = 0.7; *p* = 0.0000. In children with ATB, IP-10 correctly classified 78.8% of patients and performed better than QFT but poorer than the TST (69.7%. and 93.3%, respectively). One child with an indeterminate QFT result was correctly classified by both the TST and IP-10. Combining the IP-10 and QFT results increased the sensitivity to 84.5%.

## 4. Discussion

The present study indicates the potential of IP-10 to detect *Mtb.* infection in children in a low TB incidence country where BCG vaccination is routinely administrated at birth. We demonstrate that IP-10 level is not correlated with age and, for the first time, report a decrease in the IP-10 level during anti-tuberculosis treatment in children.

In agreement with previous studies, we show that IP-10 can distinguish *Mtb.*-infected children from uninfected children [18,19,20,22,24,25,26,27]. Unlike some other reports, we observed increased *Mtb.* antigen-stimulated expression of IP-10 only in the infected children [22,26]. While the significant differences in IP-10 levels between *Mtb.-*infected and uninfected contact tracing referrals have already been reported by others [12,16,20,22,25,26], IP-10 has not been extensively investigated in symptomatic children with a clinical suspicion of ATB. As far as we know, this study is the first to show that IP-10 can identify *Mtb.* infection in symptomatic children under diagnosis for TB in a low-endemic setting. Previous studies by Petrone et al. and Sudbury et al. were performed in high-incidence countries [28,29]. Petrone et al. reported contradictory results compared to our study but employed unstimulated plasma, which may have accounted for the observed differences [28]. In line with our report, Sudbury et al. noted significantly higher IP-10 levels in ATB children than in symptomatic children with other final diagnoses [29]. However, the number of ATB patients was low (n = 5 as compared to n = 33 in this report).

Although the *Mtb.* antigen-stimulated IP-10 level was higher in children with ATB than in the TBI group (*p* = 0.047), no power of IP-10 to discriminate between ATB and TBI has been demonstrated in ROC analyses. Our findings stay in line with the majority of previous reports in adults and children [18,21,30,31].

We observed good agreement between IP-10 and both the TST and QFT. Moreover, combining the IP-10 and IFN-γ results improved the QFT sensitivity in patients with ATB, in whom IGRAs’ performance remains suboptimal. The benefits of combining biomarker approaches in childhood TB have already been documented [16,17,18,24,27,31]. However, several studies conducted in a high endemic setting and children with ATB have yielded conflicting results, warranting further research in this field [19,32,33].

On par with most evidence in children, we did not demonstrate a relevant correlation of IP-10 with age [19,24,26,31]. Additionally, in line with a previous study from our center, we did not observe compromised IFN-γ responses in younger children [9].

Furthermore, the present study is, as far as we know, the first to report a decrease in IP-10 levels during anti-TB treatment in children. While a decline in plasma IP-10 level after successful anti-TB treatment has been demonstrated in adults, it has not been broadly investigated in children [21,22,34,35,36,37]. Nausch et al. analyzed a spectrum of cytokines before and during anti-TB treatment and reported a significant reduction only in the expression of IFN-γ with no change in IP-10 level [22]. In contrast, we observed a significant decline in IP-10 concentration with no decrease in IFN-γ level. This decline in IP-10 expression was detected only in children with ATB, who had the highest concentrations at baseline. We assume that differences in time to follow-up visit (median 69 days in our study versus 90 days in the other report) could explain the discrepancies related to IFN-γ but not IP-10. Our results support the previous study by Wergeland et al., who demonstrated a decrease in IP-10 level in adult patients with ATB already after 6–12 weeks of treatment [21].

Another strength of the present study is the control group consisting of healthy children with a population risk of TB infection. Numerous studies have included TB contacts, children with respiratory tract infections, or healthy adults in the control group [18,20,22,25,26,28,31]. We consider our approach reasonable for several reasons. First, *Mtb.* infection cannot be definitively excluded in children recently exposed to TB. Second, IP-10 can be induced during diverse inflammatory diseases including infections [38,39]. Third, a direct comparison of IP-10 levels solely between children provides relevant information on its expression in this age group. Taken together, including healthy children in the control group might have limited the risk of bias in the present report.

The main limitation of our study is the relatively small sample size. This is not uncommon in studies conducted in children in TB low-burden areas and has been previously reported [20,31]. Furthermore, our results might be biased by the potential false classification of participants. Similar to others, we applied stringent criteria for ATB diagnosis to limit the risk of misclassification [20,22,26]. The rate of culture-positive cases reflected other observations in children, and no significant differences in IP-10 or IFN-γ level were demonstrated between ATB children with and without a positive culture result [40]. Therefore, we assume that the classification of ATB patients was correct; however, the possibility of misclassification cannot be truly excluded.

Furthermore, the TBI group in the present study comprises children with either positive TST or QFT results. Since the TST false-positivity rate does not exceed 8.5% in individuals vaccinated in infancy and only two participants with TST^−^/QFT^+^ discordance presented with QFT results relatively close to the cut-off, we believe that this cannot account for the large proportion of discordant TBI patients in our report [4]. Similar to others, we observed higher IP-10 levels in children with TST^+^QFT^+^ TBI than in children with discordance [17,20]. Interestingly, contrary to Petrucci et al., no differences were noted between children with different discordant result constellations. Since children with discordance constitute a significant management problem in pediatrics, this is an area that merits further research on fluctuations in TB biomarkers during different stages of *Mtb.* infection.

Applying QFT negativity as a criterion for *Mtb.-*uninfected groups might have caused selection bias and rendered the comparison of the specificity of IP-10 and QFT impossible. Furthermore, we neither assessed the severity of TB nor were children with the most severe forms of disease included. Additionally, the children with ATB were significantly older than the children in the other groups. Therefore, IP-10 performance in children with severe TB and the youngest children with ATB may differ from that presented here. Routine testing for HIV was not performed as part of this study, but HIV test results were recorded if available and no cases were reported. Notably, HIV incidence in Poland is low (<1/100,000) with marginal TB-HIV coexistence [41]. Nonetheless, the performance of IP-10 in HIV-infected children in our setting remains uncertain.

In conclusion, our data demonstrate that *Mtb.-*specific IP-10 measurement has potential as a diagnostic biomarker of childhood TB. In particular, IP-10 seems to be a valid surrogate marker for IFN-γ in the assays based on the QFT platform. Our data show that IP-10 alone does not allow for the distinction between ATB and TBI. This is the first study with a clinical approach to TB diagnosis in children that shows good performance of IP-10 in identifying *Mtb*. infection in TB contact referrals and children with clinical suspicion of TB in a low TB-endemic country. Additionally, this is the first report demonstrating a significant decline in IP-10 levels during anti-TB chemotherapy in children. Collectively, these findings suggest that IP-10 has the potential to become a TB marker in the pediatric population and may be used in treatment monitoring.

## Figures and Tables

**Table 1:** Demographic and clinical characteristics of the study participants.

	Total n = 225 (%)	ATB n = 33 (%)	TBI n = 48 (%)	TB Contact n = 83 (%)	non-TB n = 20 (%)	Controls n = 41 (%)
Gender						
female	110 (48.9)	18 (54.5)	23 (48)	36 (43.4)	12 (60)	21 (51.2)
male	115 (51.1)	15 (45.5)	25 (52)	47(56.6)	8 (40)	20 (48.8)
age (yrs), median (IQR)	6 (3–11)	13 (7–16)	7 (4.5–11)	4 (2–8)	7 (5–10.5)	6 (1–11)
≤5 y	84 (37.3)	6 (18.2)	12 (25)	45 (54.2)	4 (20)	17 (41.5)
0–2 y	44 (19.6)	3 (9.1)	7 (14.6)	20 (24.1)	2 (10)	12 (29.3)
2–5 y	40 (17.8)	3 (9.1)	5 (10.4)	25 (30.1)	2 (10)	5 (12.2)
>5 y	141 (62.7)	27 (81.8)	36 (75)	38 (45.8)	16 (80)	24 (58.5)
5–10 y	71 (31.6)	4 (12.1)	20 (41.7)	24 (28.9)	10 (50)	13 (31.7)
>10 y	70 (31.1)	23 (69.7)	16 (33.3)	14 (16.9)	6 (30)	11 (26.8)
BCG-vaccinated						
yes	217 (96.4)	30 (91)	48 (100)	80 (96.4)	19 (95)	40 (97.6)
no	3 (1.3)	1 (3)	-	-	1 (5)	1 (2.4)
not recorded	5 (2.2)	2 (6)	-	3(3.6)	-	-
Confirmed TB	15 (6.7)	15 (45.4)	-	-	-	-
microscopy	7 (3.1)	7 (21.1)	-	-	-	-
PCR	12 (5.3)	12 (36.4)	-	-	-	-
culture	9 (4)	9 (27.3)	-	-	-	-
TST results						
positive	72 (32)	28 (84.8)	41 (85.4)	-	3 (15)	-
negative	99 (44)	2 (6)	5 (10.4)	77 (92.8)	15 (75)	-
not performed	54 (24)	3 (9.1)	2 (4.2)	6 (7.2)	2 (10)	41 (100)
QFT results						
positive	56 (24.9)	23 (69.7)	33 (68.6)	-	-	-
negative	167 (74.2)	9 (27.3)	15 (31.3)	82 (98.8)	20 (100)	41 (100)
indeterminate	2 (0.9)	1 (3)	-	1 (1.2)	-	-
TST+/QFT−	45 (20)	19 (57.6)	26 (54.2)	-	-	-
TST+/QFT−	23 (10.2)	8 (24.2)	15 (31.3)	-	3 (15)	-
TST−/QFT+	6 (2.7)	1 (3)	5 (10.4)	-	-	-
TST−/QFT−	1 (0.4)	1 (3)	-	-	-	-

Definition of abbreviations: ATB—active tuberculosis; TBI—tuberculous infection; TB—tuberculosis; IQR—interquartile range; BCG—bacillus Calmette–Guérin; PCR—polymerase chain reaction; TST—tuberculin skin test; QFT—QuantiFERON TB Gold-In Tube.

**Table 2:** IP-10 concentrations in QFT supernatants.

	IP-10 Unstimulated Median (IQR) pg/mL	IP-10 Mitogen Stimulated Median (IQR) pg/mL	IP-10 *Mtb*. Antigen-Stimulated Median (IQR) pg/mL	*p* ^a^-Value
ATB	257 (158–637)	18,963 (10,857–26,266)	7930 (1194–36,968)	<0.001
TBI	335 (198–538)	25,456 (11,330–43,298)	2250 (282–9168)	<0.001
TB contact	208 (133–328)	4874 (4056–20,811)	82 (21–188)	>0.05
Non-TB	433 (266–721)	20,026 (1329–38,490)	155 (16–484)	>0.05
Control	387 (244–610)	788 (424–28,501)	143 (12–582)	>0.05

Definition of abbreviations: QFT—QuantiFERON TB Gold-In Tube; IQR—interquartile range; *Mtb*.—*Mycobacterium tuberculosis*; ATB—active tuberculosis; TBI—tuberculous infection; TB—tuberculosis. ^a^ compares the IP-10 values after *Mtb*. antigen stimulation to the IP-10 unstimulated values.

**Table 3:** Receiver operating characteristic analyses of plasma IP-10 for *Mtb*. infection and disease.

	Cut-Off pg/mL	AUC (95% CI)	*p*-Value	Sensitivity (95% CI)	Specificity (95% CI)
Total cohort:					
ATB vs. control	>1084.5	0.935 (0.836–0.983)	<0.0001	93.3 (68.1–99.8)	87.8 (73.8–95.9)
TBI vs. control	>1498.1	0.977 (0.906–0.998)	<0.0001	88.5 (69.8–97)	97.6 (87.1–99.9)
ATB+TBI vs. control	>1084.5	0.961 (0.894–0.991)	<0.0001	92.68 (80.1–98.5)	87.8 (73.8–95.9)
ATB vs. TBI	≤2593.3	0.556 (0.393–0.711)	0.5692	44.4 (13.7–78.8)	88.5 (69.8–97.6)
Clinical suspicion of TB:					
ATB vs. RTI	>626.8	0.921 (0.746–0.990)	<0.0001	90.9 (58.7–99.8)	93.3 (68.1–99.8)
Contact tracing referrals:					
Mtb.-infected vs. TB contact	>761	0.999 (0.963–1.000)	<0.0001	100 (88.1–100)	98.7 (92.9–100)

Definition of abbreviations: Mtb.—*Mycobacterium tuberculosis*; ATB—active tuberculosis; TBI—tuberculous infection; TB—tuberculosis; AUC—area under the ROC curve; 95% CI—95% confidence interval.

## Data Availability

The datasets used and analyzed during the current study are available from the corresponding author upon reasonable request.

## Abbreviations

ATB	Active tuberculosis
IFN-γ	Interferon gamma
IGRAs	Interferon-gamma release assays
TBI	Tuberculous infection
Mtb.	Mycobacterium tuberculosis
PTB	Pulmonary tuberculosis
TB	Tuberculosis
